# An Integrated Analysis of the Role of Gut Microbiome-Associated Metabolites in the Detection of MASH-Related Cirrhosis

**DOI:** 10.3390/metabo14120681

**Published:** 2024-12-04

**Authors:** Feixiang Xiong, Xuejie Zhang, Yuyong Jiang, Peipei Meng, Yang Zhou, Xiaomin Ji, Jialiang Chen, Tong Wu, Yixin Hou

**Affiliations:** Center of Integrative Medicine, Beijing Ditan Hospital, Capital Medical University, No. 8 Jing Shun East Street, Beijing 100015, China; xiongfeixiang99@gmail.com (F.X.); jiangyuyong100015@gmail.com (Y.J.);

**Keywords:** Prevotella, linolenic acid, MASH, gut microbiota, gut metabolites

## Abstract

Background and aim: The prevalence and adverse outcomes of metabolic dysfunction associated with steatotic liver disease (MAFLD) are increasing. The changes in the gut microbiota and metabolites associated with metabolic dysfunction-associated steatohepatitis (MASH) are regarded as an essential part of the progression of MAFLD. This study aimed to identify the gut microbiota and metabolites involved in the development of MAFLD in patients. Method: This study enrolled 90 patients (healthy controls, HC: *n* = 30; MASH: *n* = 30; MASH-related cirrhosis, MC: *n* = 30), and their fecal samples were collected for 16S rRNA sequencing and non-targeted LC–MS/MS metabolomics analysis. Data preprocessing and statistical analyses were performed using QIIME2 software, Pynast, QIIME2 package, Progenesis QI, and R program. Results: The abundance of Prevotellaceae at the family level and Prevotella at the genus level was lower in the MASH and NC samples than in the HC samples. Both Prevotellaceae and Prevotella showed the strongest correlation with MASH progression via random forest analysis. Untargeted metabolomics was used to quantitatively screen for discrepant metabolites in the stool samples from the three groups. Linolenic acid (LA)-related metabolite levels were significantly lower in MASH and NC samples. Associations between Prevotella- or LA-related metabolites and liver function were discovered. A high abundance of Prevotella was associated with LA-related metabolites and MASH. Conclusion: This study identified that gut microbiota and metabolites are associated with MASH-related metabolic dysfunction. LA and Prevotella are depleted during MASH progression, and additional supplementation with Prevotella may be a potential strategy for the future treatment of MAFLD.

## 1. Introduction

Metabolic dysfunction is associated with steatotic liver disease (MAFLD), a new definition of fatty liver officially proposed by an international expert group in 2020 after non-alcoholic fatty liver disease [1], which affects at least one-quarter of the adult population worldwide [2]. MAFLD is closely associated with metabolic diseases and its complications and has rapidly become one of the leading causes of hepatocellular carcinoma (HCC) and cirrhosis in Western countries [3]. Metabolic dysfunction-associated steatohepatitis (MASH) is a subtype of MAFLD characterized by inflammatory infiltration in the liver. Research indicates that the incidence of MASH is growing rapidly in China, with future rates potentially increasing by 15–56% and liver-related mortality more than doubling [4]. Given the increasing burden of both MASH and MASH-induced cirrhosis in the liver, understanding the pathogenesis of MASH and cirrhosis and implementing effective treatments are current research priorities.

Recent studies have increasingly identified a close association between gut microbiota and the occurrence of MASH and liver cirrhosis. Alterations in the gut microbiota may contribute to the onset of MASH and its progression to cirrhosis. The gut microbiota can influence the development of MASH through various mechanisms, including changes in the metabolism of substances such as bile acids, trimethylamine (TMA), and trimethylamine N-oxide (TMAO) [5,6], and by affecting the immune functions of the gut and liver, leading to the production of pro-inflammatory cytokines [7,8]. In patients with liver cirrhosis, the gut microbiota differs from that of healthy individuals, showing a decrease in *Bacteroidetes* and an increase in *Proteobacteria* and *Firmicutes* [9]. Other studies have observed greater variations in levels of *Enterobacteriaceae* in patients [10]. The gut microbiome also can serve as a non-invasive diagnosis marker for MAFLD. Research has shown that the area under the curve (AUC) using the gut microbiome for diagnosing MASH-related cirrhosis reached 0.91 in the training cohort and 0.95 in the validation cohort [11]. In addition, the gut microbiota may represent a potential therapeutic approach for MAFLD. Studies have shown that exogenous supplementation with *Bacteroides ovatus* and *Parabacteroides* can alleviate MASH [12,13], while supplementation with *Lactobacillus acidophilus* can produce valeric acid to treat MAFLD-associated HCC [14].

Alterations in the gut microbiota also affect changes in gut metabolites, which are closely related to disease severity and can serve as biomarkers to differentiate between healthy individuals and those with MAFLD [15,16]. Compared with healthy individuals, morbidly obese females with MASH exhibit increased levels of diglycerides, lysodiol-phosphate-ethanolamine, phosphatidylinositol, and phosphatidylethanolamine, whereas levels of acylcarnitine, linoleic acid (LA), and most sphingolipids decrease [17]. The increased metabolism of bile acids and their derivatives exacerbates the development of MAFLD [18]. Changes in gut metabolites can further distinguish cirrhotic patients from healthy individuals [19], with significant alterations observed in bile acids [20], short-chain fatty acids [21], and other metabolites in cirrhotic patients compared with healthy controls [22].

Although previous studies have identified characteristic differences in gut microbiota and metabolites between patients with MASH and healthy individuals, there is currently a lack of research explaining the microbiota that can potentially benefit patients with MASH. Therefore, we performed an integrated analysis of metabolomics and 16S rDNA sequencing of paired fecal samples from patients with progressive MASH and MASH-related cirrhosis. A variety of bioinformatic analyses have been used to identify healthy individuals—the MASH-cirrhosis disease progression axis—and optimize the gut microbiota and metabolites, which are beneficial to liver function. This study provides a basis for future treatment strategies for patients with MASH.

## 2. Methods

### 2.1. Study Cohort

Ninety patients were included in this study: 30 healthy controls (HC), 30 patients with MASH, and 30 patients with MASH-related liver cirrhosis (NC). Fecal samples from these patients were collected during outpatient visits or hospitalizations. All the samples were stored at −80 °C immediately after collection. Fecal samples from 90 patients were processed together for 16S rDNA sequencing and untargeted metabolomic analysis, with the following inclusion criteria: (1) age > 18 years and (2) a diagnosis meeting the criteria for MASH and MASH-related liver cirrhosis. The exclusion criteria were as follows: (1) coexisting hepatitis B virus (HBV) infection, hepatitis C virus (HCV)infection, or other viral hepatitis; (2) coexisting liver diseases due to other causes, such as alcohol, drugs, genetic factors, or autoimmune disorders; (3) coexisting HCC or tumors at other sites; and (4) coexisting HIV infection.

### 2.2. Sample Preparation

Samples stored at −80 °C were thawed at room temperature. Thirty mg of sample was added to a 1.5 mL Eppendorf tube with 98.00% of L-2-chlorophenylalanine dissolved in methanol as internal standard, and the tube was vortexed for 10 s. Subsequently, 400 μL of ice-cold mixture of methanol and acetonitrile (2/1, *v*/*v*) was added, the mixtures were vortexed for 1 min, and the whole samples were extracted by ultrasonic for 10 min in an ice-water bath, stored at −40 °C for 24 h. The extract was centrifuged at 4 °C (12,000× *g* rpm) for 10 min. The supernatant (150 μL) from each tube was collected using a crystal syringe, filtered through a 0.22 μm microfilter and transferred to an LC vial. The vials were stored at −20 °C until LC–MS analysis. Quality control (QC) samples were prepared by mixing aliquots of all samples to form a pooled sample.

### 2.3. 16S rDNA Extraction and Amplification

Total genomic DNA was extracted using a MagPure Soil DNA LQ Kit (Magan, Guangzhou, China) following the manufacturer’s instructions. DNA concentration and integrity were measured using NanoDrop 2000 (Thermo Fisher Scientific, Waltham, MA, USA) and agarose gel electrophoresis. The extracted DNA was stored at −20 °C until further processing. The extracted DNA was used as a template for PCR amplification of bacterial 16S rRNA genes using barcoded primers and Takara Ex Taq (Takara, Tokyo, Japan). For bacterial diversity analysis, V3-V4 variable regions of 16S rRNA genes were amplified with universal primers 343F (5′-TACGGRAGGCAGCAG-3′) and 798R (5′-AGGGTATCTAATCCT-3′) [23].

### 2.4. Library Construction and Sequencing

The amplicon quality was visualized using agarose gel electrophoresis. PCR products were purified using AMPure XP beads (Agencourt, Indianapolis, IN, USA) and amplified in another round of PCR. After purification with AMPure XP beads, the final amplicon was quantified using a Qubit dsDNA Assay Kit (Thermo Fisher Scientific, Waltham, MA, USA). The concentrations were adjusted for sequencing. Sequencing was performed on an Illumina NovaSeq 6000 with 250 bp paired-end reads (Illumina Inc., San Diego, CA, USA; OE Biotech Company, Shanghai, China).

### 2.5. LC–MS/MS Analysis

The metabolomic data analysis was performed by Shanghai Luming Biological Technology Co., Ltd. (Shanghai, China). An ACQUITY UPLC I-Class Plus (Waters Corporation, Milford, CT, USA) fitted with a Q-Exactive mass spectrometer equipped with a heated electrospray ionization (ESI) source (Thermo Fisher Scientific, Waltham, MA, USA) was used to analyze the metabolic profiles in both positive and negative ion modes. An ACQUITY UPLC HSS T3 column (1.8 μm, 2.1 × 100 mm) was employed in both positive and negative modes. The binary gradient elution system consisted of (A) water (containing 0.1% formic acid) and (B) acetonitrile, and separation was achieved using the following gradient: 0.00 min, 5% B; 2 min, 5% B; 4 min, 30% B; 8 min, 50% B; 10 min, 80% B; 14 min, 100% B; 15 min, 100% B; 15.1 min, 5%, and 16 min, 5%B. The flow rate was 0.35 mL/min and the column temperature was 45 °C. All the samples were kept at 10 °C during the analysis. The injection volume was 2 L.

The mass range was 100–1000. The resolution was set to 70,000 for the full MS scans and 17,500 for the HCD MS/MS scans. The collision energies were set to 10, 20, and 40 eV. The mass spectrometer operated as follows: spray voltage, 3800 V (+) and 3200 V (−); sheath gas flow rate, 35 arbitrary units; auxiliary gas flow rate, 8 arbitrary units; capillary temperature, 320 °C; auxiliary gas heater temperature, 350 °C; S-lens RF level, 50.

### 2.6. Data Analysing

Data preprocessing and statistical analysis were performed using QIIME2 software (2020.11) [24], PyNAST, QIIME2 package, Progenesis QI V2.3 (Nonlinear, Dynamics, Newcastle, UK), and R program (version 4.4.1).

## 3. Results

### 3.1. Baseline Characteristics

Ninety patients were included in the study. The discovery cohort was divided into three groups: HC (*n* = 30), MASH (*n* = 30), and NC (*n* = 30). Each stool sample was subjected to 16 S rDNA sequencing and non-targeted LC–MS/MS metabolomic analysis. The sex distribution among MASH patients and HC was similar, although there were a higher proportion of males among MASH patients, accounting for 80%. Both MASH and NC patients exhibited elevated levels of ALT, AST, and ALP compared with HC, indicating abnormal liver function. Additionally, body mass index, total cholesterol, and triglyceride levels were significantly higher in MASH and NC patients than in normal controls, suggesting disturbances in lipid metabolism. These baseline characteristics indicated that patients with MASH and NC had abnormal liver function and disrupted lipid metabolism (Table 1).

### 3.2. Variation in Gut Microbial Abundance Across Different Groups

**Figure 1:**
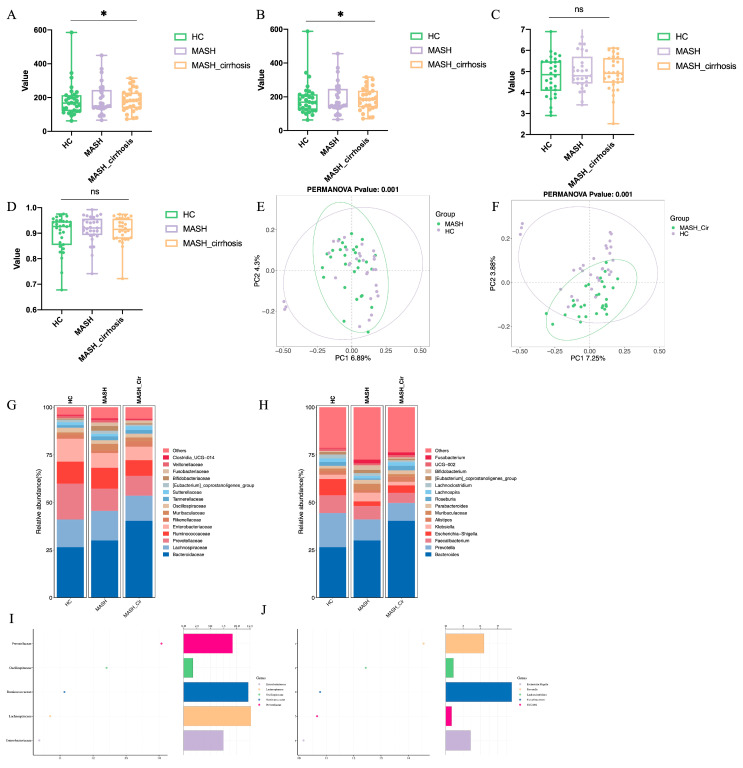
(**A**) ACE index boxplot in three groups; (**B**) Chao1 index boxplot in three groups; (**C**) Shannon index boxplot in three groups; (**D**) Simpson index boxplot in three groups; (**E**) Principal Coordinates Analysis (PCoA) between MASH and HC patients; (**F**) PCoA analysis between NC and HC patients; (**G**) the abundance of the top 15 gut microbiota in three groups at family level; (**H**) the abundance of the top 15 gut microbiota in three groups at genus level; (**I**) the importance of decreased gut microbiota in three groups at the family level via RF analysis; (**J**) the importance of decreased gut microbiota in three groups at the genus level via RF analysis. *, *p* < 0.05; ns, *p* > 0.05. Abbreviations: HC, healthy control; MASH, nonalcoholic steatohepatitis; NC, nonalcoholic steatohepatitis cirrhosis; RF, random forest.

To compare the richness and diversity of the gut microbiota of the three groups, α-diversity was assessed using ACE, Chao1, Shannon, and Simpson indices for three groups. The results indicated that the ACE and Chao1 indices were significant in all three groups, whereas the Shannon and Simpson indices were not (Figure 1A–D). β-diversity was analyzed using Principal Coordinates Analysis (PCoA), revealing significant differences in gut microbiota distribution between MASH and NC patients compared with normal HC (*p* = 0.001) (Figure 1E,F). The α-diversity and β-diversity results demonstrated that gut microbiota could have an effect on MAFLD.

At the family level, the most abundant taxa across the three groups were *Bacteroidaceae* (HC: 28.46%, MASH: 30.07%, NC: 40.35%), *Lachnospiraceae* (HC: 14.58%, MASH: 15.52%,NC: 13.21%), *Ruminococcaceae* (HC: 18.84%, MASH: 11.66%, NC: 10.42%), *Prevotellaceae* (HC: 11.59%, MASH: 10.94%, NC: 8.23%), and *Enterobacteriaceae* (HC: 11.95%, MASH: 7.77%, NC: 7.03%) (Figure 1G). The most abundant taxa at the genus level were *Bacteroides* (HC: 26.46%, MASH: 30.07%, NC: 40.45%), *Faecalibacterium* (HC: 18.00%, MASH: 11.02%, NC: 0.38%), *Prevotella* (HC: 9.26%, MASH: 7.07%, NC: 5.29%), and *Escherichia–Shigella* (HC: 8.58%, MASH: 2.37%, NC: 3.97%) (Figure 1H).

Given that MASH and NC are increasing global health burdens, fecal microbiota transplantation may be a potential treatment option for patients with MASH. Therefore, identifying the key bacterial taxa associated with progression of MASH is crucial. This study identified the top 15 gut microbiota taxa at the family and genus levels that were reduced in patients with MASH and NC compared with HC. The results showed that the abundances of *Enterobacteriaceae*, *Lachnospiraceae*, *Oscillospiraceae*, *Ruminococae*, and *Prevotellaceae* at the phylum level in patients with MASH and NC were significantly lower than those in the HC group. (Figure 1G). At the genus level, *Escherichia–Shigella*, *Prevotella*, *Lachnoclostridium*, *Faecalibacterium*, and *UCG-002* were all decreased in abundance in patients with MASH and MC compared with HC (Figure 1H), with *Prevotella* especially having decreased 10% in MASH and MC compared with HC (*p* = 0.011). To further clarify the role of *Prevotella* in MASH progression, a random forest (RF) analysis was used to rank the importance of decreasing bacterial taxa in patients with MASH and NC. Both *Prevotellaceae* at the family level and *Prevotella* at the genus level showed the strongest correlation with MASH progression. (Figure 1I,J). The above results indicate that a decrease in Prevotella abundance may be involved in the development of MASH and may be a potential treatment strategy for MASH.

### 3.3. Structural Modulation of the Gut Microbiota Between MASH and NC Patients

**Figure 2:**
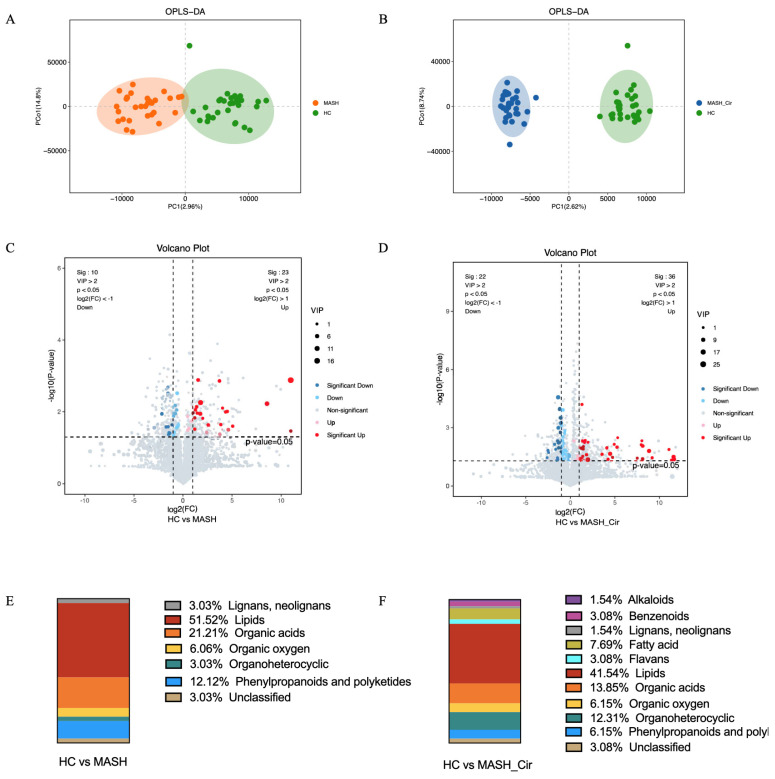
(**A**) OPLS-DA analysis between HC and MASH patients; (**B**) OPLS-DA analysis between HC and NC patients; (**C**) filtering out the metabolites identified through both univariate and multivariate statistical analyses via volcano plots in MASH patients (VIP > 2, *p* < 0.05, |log2FC| > 1); (**D**) filtering out the metabolites identified through both univariate and multivariate statistical analyses via volcano plots in NC patients (VIP > 2, *p* < 0.05, |log2FC| > 1); (**E**) the main gut metabolites between HC and MASH patients; (**F**) the main gut metabolites between HC and NC patients. Abbreviations: HC, healthy control; MASH, non-alcoholic steatohepatitis; NC, non-alcoholic steatohepatitis cirrhosis; VIP, variable importance in projection; FC, fold change.

To distinguish the differences in gut metabolites among the three groups, pairwise comparisons were conducted using OPLS-DA. The results demonstrated significant differences in gut metabolites between patients with MASH and NC compared with HC, effectively differentiating healthy individuals from patients with MASH and NC (Figure 2A,B).

Further analysis utilized both univariate tests (Mann–Whitney U test) and multivariate analysis (OPLS-DA) to identify differential metabolites between patients with MASH, NC, and healthy individuals. For OPLS-DA, metabolites with a variable importance in projection (VIP) value greater than 2 were considered significant, and volcano plot thresholds were set as follows (1. *p* < 0.05; 2. |log2FC| > 1 (fold-change)). Volcano plots displayed the differential metabolites identified through both univariate and multivariate statistical analyses (Figure 2C,D), and the metabolites are shown in Table 2.

Univariate analysis revealed different changes in the upregulation and downregulation of gut metabolites in patients with MASH and NC. In patients with MASH, 33 metabolites were identified, 23 of which were upregulated and 10 were downregulated. The major metabolite categories included lipids (51.52%), organic acids (21.21%), phenylpropanoids and polyketides (12.12%), organic oxygen (6.06%), organoheterocyclic compounds, lignans (3.03%), and neolignans (3.03%) (Figure 2E). In NC patients, 58 metabolites were identified, of which 36 were upregulated and 22 were downregulated. Major metabolite categories included lipids (41.54%), organic acids (13.85%), organoheterocyclic (12.31%), fatty acids (7.69%), organic oxygen (6.15%), phenylpropanoids and polyketides (6.15%), benzenoids (3.08%), flavans (3.08%), alkaloids (1.54%), and lignans and neolignans (1.54%) (Figure 2F).

These results indicate that gut metabolites may be present in patients with MASH and liver cirrhosis, with lipids possibly being the most significantly affected category.

### 3.4. Linolenic Acid Metabolism Promotes the Onset and Progression of MAFLD

**Figure 3:**
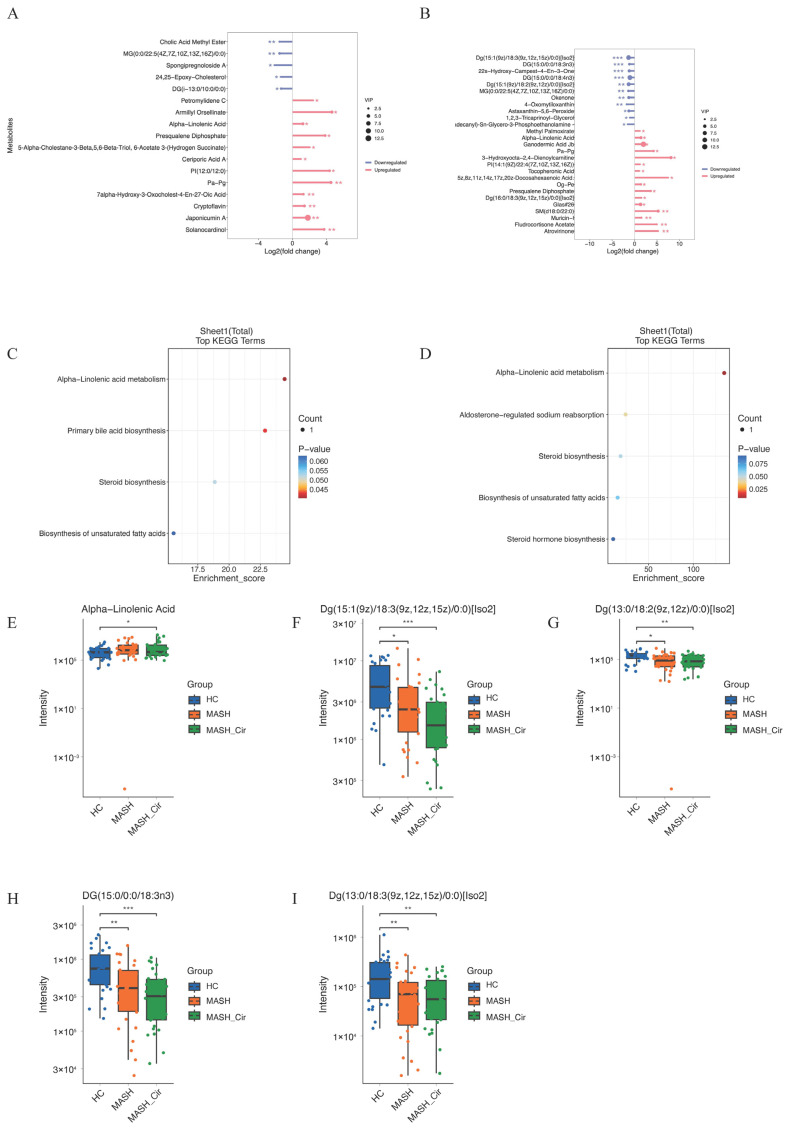
(**A**) Upregulation and downregulation of lipid gut metabolites in MASH patients; (**B**) upregulation and downregulation of lipid gut metabolites in NC patients; (**C**) the KEGG enrichment via lipid gut metabolites in MASH patients; (**D**) the KEGG enrichment via lipid gut metabolites in NC patients; (**E**–**I**) LA-related metabolites level in three groups. Abbreviations: HC, healthy control; MASH, nonalcoholic steatohepatitis; NC, nonalcoholic steatohepatitis cirrhosis; LA, linolenic acid. *, *p* < 0.05; **, *p* < 0.01; ***, *p* < 0.001.

A detailed analysis of the lipid metabolic profiles of patients in the MASH and NC groups was conducted to clarify the effects on lipid metabolism. In patients with MASH, 17 lipid metabolic products were identified, of which 12 were upregulated and 5 were downregulated. (Figure 3A). In patients with cirrhosis, 27 lipid metabolic products were identified, 16 of which were upregulated and 11 were downregulated (Figure 3B). Enrichment KEGG analysis revealed that in MASH patients, alpha-linolenic acid metabolism, Primary Bile Acid Biosynthesis, Steroid Biosynthesis, and Biosynthesis of Unsaturated Fatty Acids, with linoleic acid metabolism being the most prominent (Figure 3C). In patients with NC, the major enriched pathways included alpha-linolenic acid metabolism, Aldosterone-Regulated Sodium Reabsorption, Steroid Biosynthesis, Biosynthesis of Unsaturated Fatty Acids, and Steroid Hormone Biosynthesis, with linoleic acid metabolism being the most significant (Figure 3D). This indicates that lipid metabolism disorders are primarily linked to linoleic acid metabolism.

To clarify the metabolic status among healthy individuals, patients with MASH, and patients with cirrhosis, metabolites were screened using univariate and multivariate analyses with the following criteria: *p* < 0.05, |log2FC| > 1, and VIP > 1. The results showed that five linoleic acid-related metabolites were common to all three groups. Among these groups, alpha-linolenic acid levels increased progressively with disease progression (*p* < 0.05), while Dg(15:1(9z)/18:3(9z,12z,15z)/0:0)[Iso2] (*p* < 0.001), Dg(13:0/18:2(9z,12z)/0:0)[Iso2] (*p* < 0.01), Dg(13:0/18:3(9z,12z,15z)/0:0)[Iso2] (*p* < 0.001), and Dg(13:0/18:3(9z,12z,15z)/0:0)[Iso2] (*p* < 0.01) showed progressively decreasing levels with disease progression (Figure 3E–I), with the most pronounced decrease observed in patients with NC. This suggests that changes in the microbiome may affect LA metabolism, leading to a reduction in LA levels.

### 3.5. Relationship Between Prevotella, LA Metabolism, and Lipid Profiles

Our study found a notable decrease in *Prevotella* abundance in patients with both MASH and NC, and RF analysis identified *Prevotella* as a critical feature across the three groups. In addition, the levels of various LA metabolites significantly decreased with disease progression, suggesting that *Prevotella* spp. and LA metabolites may be related to MAFLD.

**Figure 4:**
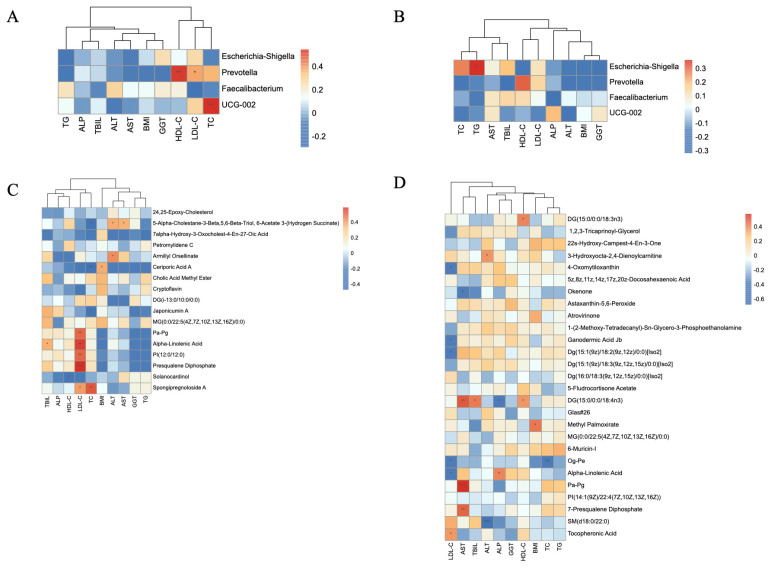
(**A**) The heatmap of the correlation between reduced gut microbiota and liver function in MASH patients; (**B**) the heatmap of the correlation between reduced gut microbiota and liver function in NC patients; (**C**) the heatmap of the correlation between lipid gut microbiota and liver function in MASH patients; (**D**) the heatmap of the correlation between lipid gut microbiota and liver function in NC patients. Abbreviations: HC, healthy control; MASH, non-alcoholic steatohepatitis; NC, non-alcoholic steatohepatitis cirrhosis. *, *p* < 0.05; **, *p* < 0.01; ***, *p* < 0.001.

Therefore, we elucidated the correlation between the gut microbiota, metabolites, and clinical indicators in patients with MASH and NC. The heatmap of the correlation between reduced gut microbiota and liver function indicators showed that in patients with MASH, *Prevotella* was closely associated with both HDL-C and LDL-C levels. The correlation between *Prevotella* and HDL-C was the most notable feature in NC patients (Figure 4A,B). The heatmap depicting the correlation between lipid metabolites and liver function indicators demonstrated that lipid metabolites were particularly associated with LDL-C levels in patients with MASH and cirrhosis (Figure 4C,D).

### 3.6. Association Between Prevotella and MASH and Cirrhosis-Associated LA

**Figure 5:**
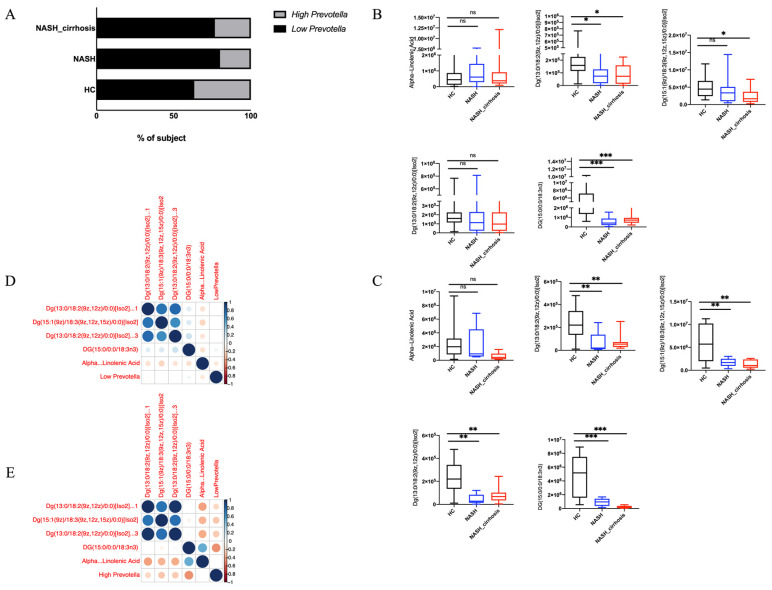
(**A**) Incidence of MASH and cirrhosis in the high- and low-abundance groups of *Prevotella* in three groups; (**B**) LA-related gut microbiota level of three groups in the low-abundance group of *Prevotella*; (**C**) LA-related gut microbiota level of three groups in the high-abundance group of *Prevotella*; (**D**) the bubble diagram of the correlation between LA-related gut microbiota and a low abundance of *Prevotellaa*; (**E**) the bubble diagram of the correlation between LA-related gut microbiota and a high abundance of *Prevotella.* Abbreviations: HC, healthy control; MASH, non-alcoholic steatohepatitis; NC, non-alcoholic steatohepatitis cirrhosis; LA, linolenic acid. *, *p* < 0.05; **, *p* < 0.01; ***, *p* < 0.001. ns, *p* > 0.05.

To explore the clinical significance of changes in *Prevotella* abundance, we divided *Prevotella* abundance into high- and low-level groups using the Youden index. The analysis showed that LA levels gradually decreased with disease progression; however, in the low-abundance group, this reduction was even more pronounced (Figure 5B,C). Finally, a heatmap showed the correlation between *Prevotella* and LA-related metabolites in the high- and low-abundance groups, indicating that higher *Prevotella* abundance was associated with more LA-related metabolites, further emphasizing the mutualistic relationship between *Prevotella* and LA metabolism (Figure 5D,E).

## 4. Discussion

This study utilized 16S rDNA sequencing to compare gut microbiota variations at the family and genus levels among three groups of individuals. RF analysis identified the *Prevotellaceae* at the family level and the *Prevotella* at genus level as the most significant, as both were notably reduced in the MASH and NC patient groups. Analysis of gut metabolites using LC–MS/MS revealed that LA metabolism was most prevalent upon KEGG pathway enrichment. A correlation was found between the microbiota, LA metabolism, and clinical indicators. Finally, we found that a high abundance of *Prevotella* was more likely to reduce the risk of MASH and NC.

*Prevotella*, an anaerobic bacterium found in the oral cavity and gut, has been linked to glucose metabolism, which is a key feature in patients with MAFLD [25]. Succinate produced by *Prevotella* can improve glucose metabolism and insulin homeostasis, thereby promoting glucose stability [26]. Additionally, *Prevotella* plays a significant role in the development of liver diseases. Studies have shown that *Prevotella* abundance is reduced in patients with primary biliary cholangitis, and exogenous supplementation with *Prevotella* can improve cholestasis and liver fibrosis via the FCR signaling pathway [27]. In chronic HBV infection, *Prevotella* abundance is notably decreased and is associated with immune suppression responses and HCC [28]. Furthermore, in patients with acute liver injury, *Prevotella* abundance gradually recovered after fecal microbiota transplantation, promoting liver damage repair by balancing Treg/Th17 cytokines [29]. These findings highlight the importance of *Prevotella* as a potential therapeutic target that influences disease occurrence and progression via various metabolic pathways.

For patients with MASH, *Prevotella* was essential for lipid metabolism. *Prevotella* may contribute to liver fat accumulation and the development of MASH by affecting iron and liver glucose metabolism [30]. Furthermore, increased *Prevotella* levels can enhance the production of metabolites related to tryptophan, tyrosine, arginine, and serine synthesis, thereby potentially improving MASH. Research has also found that in the pathogenesis of MASH, *Prevotella* is associated with 98.6% of Asians, and lower linoleic acid levels have been identified as potential biomarkers for MASH development [31]. Given that *Prevotella* can alleviate MASH, exogenous supplementation with *Prevotella* could be a potential therapeutic strategy. There are numerous therapeutic targets for *Prevotella*, which are currently believed to enhance carbon metabolism, regulate nicotinamide adenine dinucleotide (NAD), and encode some enzymes necessary for the synthesis of branched-chain amino acids (BCAA), making it a potential therapeutic site [32,33]. *Prevotella* shows considerable promise as a biomarker for diagnosing and treating MASH; our study also found that its abundance is reduced in MASH and NC patients, and high abundance is related to MASH.

LA is an essential long-chain polyunsaturated fatty acid that humans cannot synthesize and is involved in glucose homeostasis [34]. In addition, LA has been identified as a positive regulator of CD8^+^ T cells, enhancing their metabolic adaptability, preventing exhaustion, and promoting antitumor effects by stimulating memory-like phenotypes [35]. LA also exerts anti-inflammatory effects by increasing IL-12 and IFN-γ levels and decreasing IL-10 levels to treat infections [36]. LA is similarly important in the liver, with higher levels of LA inversely correlating with lower liver enzyme levels, without inducing inflammation in the liver or adipose tissue [37]. For MAFLD treatment, lifestyle modification is crucial, although such changes may only reduce weight rather than improve obesity, and appropriate supplementation with LA can ameliorate obesity [38]. Moreover, increased LA levels can improve liver fibrosis by inhibiting TGF-β signaling in hepatic stellate cells [39]. Conversely, disturbances in linoleic acid metabolism can lead to various diseases, including the transition from intrahepatic bile duct stones to intrahepatic bile duct cancer [40]. Our study found that, compared with HC, patients have significantly lower LA levels, and NC patients had even lower levels than those with MASH, consistent with previous research, suggesting a relationship between LA and the occurrence and progression of MASH.

The relationship between *Prevotella* and LA metabolism is currently underexplored; however, research has shown that both *Prevotella* and LC levels are lower in patients with allergic rhinitis than in healthy individuals [41]. Moreover, exogenous LA supplementation significantly increased *Prevotella* abundance [42]. Our study also observed this phenomenon and found that a high abundance of *Prevotella* was associated with MASH or NC progression and LA-related metabolism, highlighting the importance of *Prevotella* and LA in MAFLD. These findings suggest a reciprocal relationship between *Prevotella* and LA, warranting further research to elucidate the underlying mechanisms and to provide new perspectives for MASH treatment.

Our study found a reduction in the abundance of Prevotella spp. and abnormalities in LA metabolism. These findings provide new insights into MAFLD pathogenesis. However, this study has some limitations. First, individual differences exist, especially biases in dietary habits, age, and sex. Therefore, it is necessary to include more samples, reduce intergroup differences, and maintain consistent results. However, a sample quality inspection showed that the current sample size ensured stability of the results. In addition, further fundamental experiments are needed to validate the molecular mechanisms by which Prevotella and LA metabolism influence the occurrence of MAFLD in support of our hypothesis.

## 5. Conclusions

In summary, this study demonstrates that Prevotella and LA metabolites may be associated with MAFLD. Our data support the view that the use of microbiomes in MASH may help develop prognostic markers and new therapeutic strategies.

## Figures and Tables

**Table 1:** Baseline characteristics of the discovery cohort.

Variables	HC (*n* = 30)	MASH (*n* = 30)	MASH_cir (*n* = 30)	*p*
Gender (male)	17 (56.67)	16 (53.33)	21 (70%)	0.184
BMI	23.50 ± 2.64	27.28 ± 4.45	25.72 ± 2.45	<0.01
ALT	20.25 ± 1.85	83.23 ± 66.33	30.60 ± 1.96	<0.001
AST	18.45 ± 7.54	43.13 ± 37.15	26.81 ± 13.75	0.030
TBIL	12.25 ± 4.66	13.33 ± 9.24	12.95 ± 5.64	0.848
ALP	38.56 ± 2.86	76.85 ± 84.17	43.72 ± 3.97	0.040
GGT	42.20± 20.42	62.16 ± 38.24	49.31 ± 33.55	0.028
TC	3.21 ± 0.97	5.05 ± 0.96	6.14 ± 1.37	<0.001
TG	1.95 ± 0.79	2.39 ± 1.38	3.44 ± 1.14	0.002
HDL-C	1.59± 0.50	1.16 ± 0.37	1.31 ± 0.40	0.151
LDL-C	2.24 ± 0.65	2.98 ± 1.00	2.94 ± 0.87	0.863

HC, healthy control; MASH, nonalcoholic steatohepatitis; MASH_cir, nonalcoholic steatohepatitis cirrhosis; BMI, body mass index; TBIL, total bilirubin; ALP, alkaline phosphatase; GGT, gamma-glutamyl transpeptidase; TC, total cholesterol; TG, triglyceride; HDL-C, high-density lipoprotein cholesterol; LDL-C, high-density lipoprotein cholesterol.

**Table 2:** Fold changes of lipid metabolites based on volcano plot (with fold change threshold = 1.0). (fold changes > 1.00, VIP > 2, *p* < 0.05).

Metabolites			
	FC	Log_2_(FC)	*p*-Value
MASH			
Japonicumin A	3.498504907	1.806738515	0.005559582
Armillyl Orsellinate	24.82231517	4.633565776	0.031878631
Pa-Pg	22.99044508	4.522962491	0.009797182
Alpha-Linolenic Acid	2.332073269	1.221613116	0.03066592
Cryptoflavin	2.640620138	1.40087678	0.007388791
Solanocardinol	13.13915014	3.715800057	0.001413631
Presqualene Diphosphate	14.34843751	3.842821736	0.022500308
MG(0:0/22:5(4Z,7Z,10Z,13Z,16Z)/0:0)	0.34893865	−1.518954688	0.003097218
PI(12:0/12:0)	20.59430036	4.36417321	0.010110545
7alpha-Hydroxy-3-Oxocholest-4-En-27-Oic Acid	2.435725023	1.284351272	0.008946342
DG(i-13:0/10:0/0:0)	0.395311365	−1.338938661	0.039191307
Cholic Acid Methyl Ester	0.348688679	−1.519988571	0.002070298
5-Alpha-Cholestane-3-Beta,5,6-Beta-Triol	4.075481869	2.026970648	0.015229478
Spongipregnoloside A	0.227589493	−2.135494141	0.011625757
Petromylidene C	5.406933509	2.434810615	0.037824272
24,25-Epoxy-Cholesterol	0.375172346	−1.414374606	0.03819523
Ceriporic Acid A	2.056293433	1.040046152	0.010820446
MASH_cir			
Ganodermic Acid Jb	3.903830017	1.964890235	0.04414704
DG(15:0/0:0/18:4n3)	0.481339253	−1.054874017	0.000575077
Dg(15:1(9z)/18:3(9z,12z,15z)/0:0)[Iso2]	0.391075419	−1.354481235	2.78308 × 10^−5^
Dg(15:1(9z)/18:2(9z,12z)/0:0)[Iso2]	0.396424348	−1.334882519	0.001028793
Glas#26	2.479330218	1.309950435	0.011135105
22s-Hydroxy-Campest-4-En-3-One	0.435600561	−1.198922284	0.000114647
3-Hydroxyocta-2,4-Dienoylcarnitine	275.2966601	8.104843297	0.040231658
SM(d18:0/22:0)	38.24174411	5.257076417	0.009909678
Okenone	0.405895627	−1.300819297	0.005967872
Astaxanthin-5,6-Peroxide	0.409120172	−1.289403421	0.010708068
Alpha-Linolenic Acid	2.676509951	1.420353016	0.047350388
DG(15:0/0:0/18:3n3)	0.443553212	−1.172820903	0.000103655
Pa-Pg	18.77693701	4.230889837	0.041219105
MG(0:0/22:5(4Z,7Z,10Z,13Z,16Z)/0:0)	0.378956443	−1.39989606	0.004766514
Presqualene Diphosphate	12.0164599	3.58694003	0.023228758
Og-Pe	2.612839347	1.385618422	0.029680018
Muricin-I	2.799969582	1.485411154	0.009281978
4-Oxomytiloxanthin	0.295862044	−1.757003469	0.007122995
Dg(16:0/18:3(9z,12z,15z)/0:0)[Iso2]	2.763554004	1.466524805	0.012452688
Fludrocortisone Acetate	30.82078849	4.945831866	0.007125469
1,2,3-Tricaprinoyl-Glycerol	0.484001127	−1.046917687	0.011111422
1-(2-Methoxy-Tetradecanyl)-Sn-Glycero-3-Phosphoethanolamine	0.341339841	−1.550719278	0.047077734
Methyl Palmoxirate	2.268021703	1.181434446	0.048516936
5z,8z,11z,14z,17z,20z-Docosahexaenoic Acid	181.224821	7.501636754	0.036880092
Tocopheronic Acid	2.145309735	1.101185956	0.03727233
PI(14:1(9Z)/22:4(7Z,10Z,13Z,16Z))	2.395456438	1.260300578	0.039598994
Atrovirinone	40.11212937	5.325966649	0.003233652

## Data Availability

Data is unavailable due to privacy or ethical restrictions.
